# The relationship between self-control and learning engagement among Chinese college students: the chain mediating roles of resilience and positive emotions

**DOI:** 10.3389/fpsyg.2024.1331691

**Published:** 2024-02-20

**Authors:** Yue-Di Yang, Chi-Liang Zhou, Zi-Qing Wang

**Affiliations:** ^1^Faculty of Education, Northeast Normal University, Changchun, China; ^2^Faculty of Education, Guangxi Normal University, Guilin, China

**Keywords:** college students, learning engagement, positive emotions, resilience, self-control

## Abstract

**Background:**

As the main driver of talent cultivation in colleges and universities, the learning and development level of college students is a core indicator of the quality of talent cultivation. The current status of college students' learning has always been a heavily researched topic. However, there is a lack of academic research on the potential mechanisms of self-control about how it affects college students' learning engagement. This study explored the relationship between college students' self-control and learning engagement and the potential mechanisms underlying this relationship with reference to a large sample.

**Methods:**

A total of 765 college students from Guangxi, China, completed the self-control scale, the resilience scale, the positive emotions scale, and the learning engagement scale. SPSS 26.0 was used to conduct common method bias tests, descriptive statistics, correlation tests, and regression analyses. Structural equation modeling was constructed using AMOS 26.0, and mediation effects were tested.

**Results:**

This article mainly used questionnaires to collect data and, on this basis, examined the relationship between self-control, resilience, positive emotions, and the learning engagement of college students. The results showed that (1) self-control positively affected college students' learning engagement; (2) resilience partially mediated the relationship between self-control and college students' learning engagement; (3) positive emotions partially mediated the relationship between self-control and college students' learning engagement; and (4) resilience and positive emotions played a chain-mediating role between self-control and college students' learning engagement.

**Conclusion:**

The present study identifies the potential mechanism underlying the association between the self-control and learning engagement of college students. The results of this study have practical implications for enhancing the learning engagement of Chinese college students by increasing their psychological resources and improving the teaching of university teachers.

## 1 Introduction

In the new development stage, promoting the high-quality development of higher education and ensuring the alignment between higher education and modernization are instrumental in pushing forward the development of China's education. College students are the major targeted group in higher learning education, so their academic performance could directly reflect the quality of higher education. Learning engagement is an important predictor of academic performance and an important indicator of the quality of education and student development (Liu, 2015). At present, college students still suffer from academic procrastination, academic burnout, a lack of motivation, and a lack of learning engagement (Huang et al., 2019; Liu et al., 2022; Yang et al., 2022). Specifically, the learning engagement of today's Chinese undergraduates stands at a moderately low level, which has become a key constraint for the transformation of higher education from “big” to “strong” (Long and Ni, 2020; Guo et al., 2021). *The Implementation Opinions on the Construction of First-Class Undergraduate Programs* (hereafter referred to as the *Opinions*) issued by the Ministry of Education of the People's Republic of China (2019) suggests that learning engagement should be increased to enhance the sense of achievement that could be gained through the improvement of students' abilities and qualities when hard work has been put into studying. In this context, learning engagement refers to a state of sustained and positive affectivity performed by students concerning their academic activities in school, which would not only reflect students' learning ability but also predict their academic achievement; such engagement is also an effective way to improve poor academic performance and address high dropout rates (Vargas-Madriz and Konishi, 2021). Scholars have conducted a lot of research on the factors affecting college students' learning engagement and categorized them into internal and external ones. The internal factors mainly include learning attitudes, self-efficacy, motivation, and academic emotions, while the external factors are mainly related to classroom teaching, teachers, and teaching environment (Ahlfeldt et al., 2005; Martin, 2009; Lin et al., 2020; Wang and Wang, 2021). Several studies have shown that self-control and emotions, as individual intrinsic factors in college students, can influence the state of learning engagement so that they have some bearing on academic procrastination and burnout (D'Mello and Graesser, 2012; Chen, D. H. et al., 2021; Diotaiuti et al., 2021c; Song et al., 2023). However, there is a lack of academic research on the potential mechanisms of self-control about how it affects college students' learning engagement. Therefore, this study applies the conservation of resources theory and the extended construct theory of positive emotions to explore in depth the relationship between self-control and learning engagement among Chinese college students, focusing on the role resilience and positive emotions play in the relationship between these two, with a view to revealing the intrinsic mechanisms by which self-control affects learning engagement and to providing support for the promotion of college students' learning. It not only expands the scope of research on the factors affecting college students' learning engagement at the theoretical level but also provides practical guidance for reaching the requirements for college students' learning engagement as stated in the *Opinions*.

## 2 Theoretical background and research hypotheses

### 2.1 Theoretical background

The conservation of resources theory (COR) states that driven by evolution, human beings have always sought to acquire, protect, and build up resources that are valuable to them, viewing resource loss as a risk or stress. Even if we do not face real stress, actively building resources and increasing resource reserves are effective strategies for preventing and successfully dealing with future stress. Moreover, the more resource-rich individuals are, the more they tend to make proactive resource investment behaviors to acquire more resources, a phenomenon known as the “resource gain spiral” (Hobfoll, 1989, 2001, 2002). Accordingly, Hobfoll (2011) further refined the theory of resource conservation by proposing the concept of “resource caravans,” which refers to the accumulation and linkage of resources, suggesting that different types of resources are closely linked with one another. This theory suggests that different types of resources are closely related and can work together to drive individual behavior. Among the categories of such resources, key resources such as self-efficacy and resilience are the most stable personality traits. More recent studies have mostly analyzed the effects of single key resources such as grit, emotion management, self-efficacy, and psychological capital on learning engagement while ignoring the mechanism by which multiple key resources jointly impact learning engagement (Lei, 2022; Chen et al., 2023; Luo et al., 2023; Zhao et al., 2023). The mechanisms by which multiple resources jointly impact individual behavior deserve further exploration.

Based on the extended construct theory of positive emotions, positive emotions are effective with regard to expanding an individual's cognitive and action paradigms (Fredrickson, 2001). Positive emotional experiences not only reflect an individual's wellbeing but also contribute to an individual's growth and development, thus generating positive value for an individual's thinking and action (Fredrickson and Branigan, 2005).

### 2.2 The relationship between self-control and learning engagement

Learning engagement refers to the willingness to learn, participation, concentration, and related affective states that learners invest in the learning process (Pike et al., 2011), while college students' learning engagement refers to the time and energy that college students invest in educational activities, as well as the support and conditions provided by the school to promote students' engagement in learning (Kuh, 2009). Self-control, as a stable personality trait, affects learning engagement (Wei, 2023). Self-control refers to an individual's ability to control his or her own internal responses, to mentally inhibit erroneous or unacceptable behavioral intentions, and to behaviorally restrain his or her performance of the corresponding behaviors (Tangney et al., 2004; De Ridder et al., 2012). Resource conservation theory suggests that self-control is an important resource that can help people achieve their goals and that in a state of self-depletion; students tend to perform their tasks more poorly (Minda and Rabi, 2015). According to the self-control model developed by Hofmann et al. (2009), self-control can be categorized into control systems and impulse systems. Individuals with high self-control are able to prevent impulsive behaviors by reducing conflicts between their behaviors and goals through planning and active control (Hofmann et al., 2014). Self-control entails the depletion of an individual's psychological resources, which, when the depletion reaches a certain extent, may trigger a failure in subsequent behavior (Baumeister et al., 2007). Learning engagement is a typical self-control dilemma, and almost all students experience conflicts between their academic goals, which are associated with long-term benefits (e.g., better career prospects, higher income levels, or higher social status), and their non-academic goals, which are goals that entail short-term benefits (e.g., stress relief or pleasure) (Duckworth et al., 2019). Research has shown that individuals' levels of self-control and learning engagement are significantly positively correlated and that good self-control is one of the most important factors affecting students' academic achievement; furthermore, it plays a crucial bridging role in the process of academic development, which significantly enhances academic self-efficacy and reduces students' procrastination behaviors in the learning process (Zhou and Bi, 2017; Chen et al., 2018; Zhu et al., 2018; Chen, D. H. et al., 2021; Song et al., 2023). Accordingly, we proposed the following hypothesis:

**Hypothesis 1:** There is a significant positive correlation between self-control and learning engagement.

### 2.3 The mediating role of resilience

Resilience, which has also been referred to as psychological elasticity, toughness, resilience, and resistance, represents the ability of an individual to adapt to adversity and setbacks through flexible adjustment, which takes the form of good adaptability on the part of the individual when facing adversity, trauma, or major stressful events (Lazarus, 1993; Windle, 2011). As an important psychological resource, psychological resilience represents an individual's positive coping and adaptation in the face of difficulties or a difficult situation, and it thus plays a protective role in both emotion and behavior (Yu and Zhang, 2005; Sun et al., 2013). Researchers have shown that resilience and self-control are significantly positively correlated, that individuals with resilience exhibit high self-control, and that good self-control is conducive to the development of individuals' levels of resilience (Brown, 2015; Zheng and Xu, 2022). College students' resilience, self-improvement, and health resilience can significantly and positively predict learning engagement, and resilience can positively contribute to learning engagement through factors such as positive parenting and perceived classroom climate (Zhang et al., 2008, 2022). We therefore proposed the following hypothesis:

**Hypothesis 2:** Resilience mediates the relationship between self-control and learning engagement.

### 2.4 The mediating role of positive emotions

The extended construct theory of positive emotions is due to the claim that positive emotions can promote students' cognitive development and increase their interest in learning, as well as construct lasting personal resources and promote learning engagement (Goetz et al., 2008; Roorda et al., 2017). Pekrun (2006) argued that positive high-arousal emotions increase learners' effort and learning engagement and that positive emotions can enable learners to experience a good sense of mastery, which leads to increased willingness to actively construct knowledge and more learning engagement; in contrast, persistent negative emotions can disengage learners from the learning task (D'Mello and Graesser, 2012). In response to adversity and stress, an individual's self-control resources may be weakened, thus leading to a failure of self-control. Individuals who experience more positive emotions can effectively replenish their self-control resources when those individuals are in a state of depletion (Park et al., 2016; Shen et al., 2021). Meanwhile, individuals who experience more positive emotions carefully consider various types of detailed information before taking action and are less likely to make impulsive decisions (Boda and Sunitha, 2018), thus preventing the low levels of learning engagement triggered by self-control failures. Researchers have shown that people with more self-control resources experience more positive emotions and fewer negative emotions (Hofmann et al., 2014), i.e., that the relationship between self-control and positive emotions is positive. Based on these results, we formulated the following hypothesis:

**Hypothesis 3:** Positive emotions mediate the relationship between self-control and learning engagement.

### 2.5 The chain mediation of resilience and positive emotions

Researchers have found that as individuals with the ability and characteristics necessary to cope with life adversities, college students who exhibit higher psychological resilience are supposed to exhibit higher cognition and better levels of emotional control in response to setbacks (Li N. et al., 2023). According to the extended construct theory of positive emotions, positive emotions can promote further positive emotions in the process of expanding and constructing resources (Fredrickson, 2001). Resilience, as a form of positive psychological capital, is significantly and positively correlated with positive emotions, and high-resilience individuals have more positive attitudes and emotions (Wang and Wang, 2013; Feldman et al., 2018). Positive emotions help individuals remain optimistic in the face of stress and setbacks and maintain lower stress levels in the face of adversity (Bajaj and Pande, 2016). The resource conservation theory also suggests that individuals who possess one resource also have access to other resources (Cao and Qu, 2014), suggesting that college students with higher levels of resilience should also experience more positive emotions and that resilience and positive emotions, which are key resources, jointly help college students achieve better performance in their academic behaviors. We therefore proposed the following hypothesis:

**Hypothesis 4:** Resilience and positive emotions play a chain mediating role in the relationship between self-control and learning engagement.

**Figure 1:**
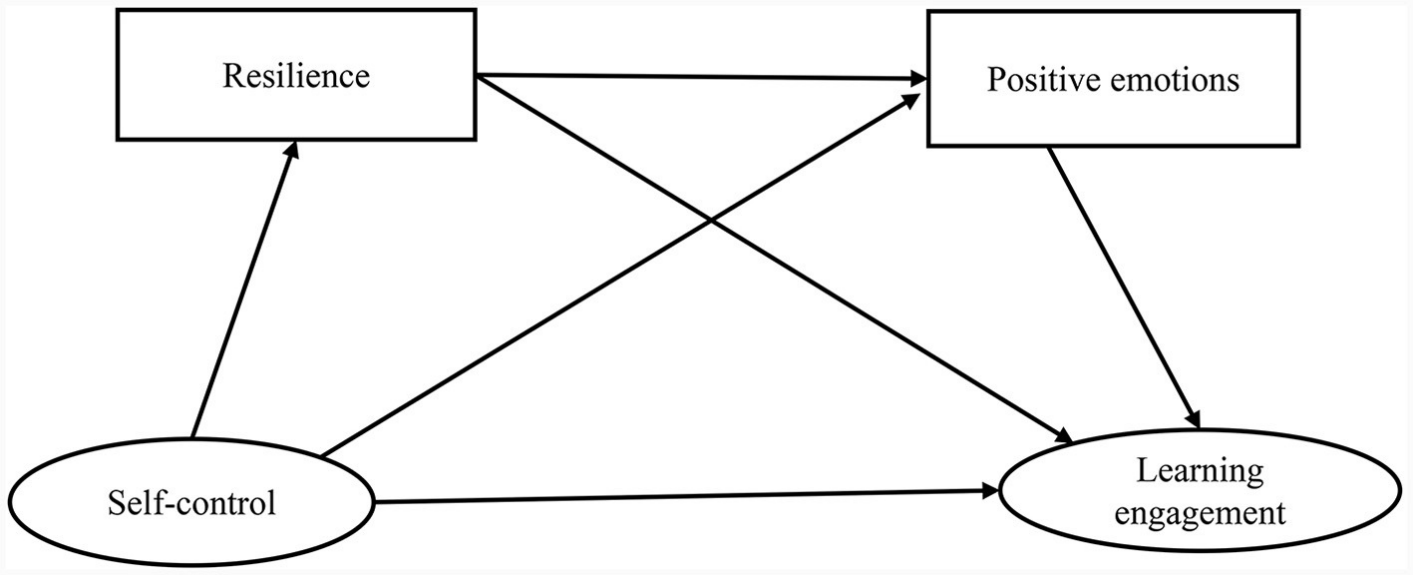
Hypothesized research model.

The present study intends to examine the relationship between self-control and learning engagement among college students and the mediating role of resilience and positive emotions in it. Synthesizing the above research hypotheses, a chain mediation model was constructed, as shown in Figure 1.

## 3 Materials and methods

### 3.1 Participants and procedure

The sample used in this study was selected from college students at Guangxi Normal University, Guilin University of Electronic Technology, Guilin University, and Nanning Normal University. Administrators and teachers from these schools were contacted, and the questionnaire was distributed from 21 to 28 September 2023, via the Questionnaire Star platform. Approximately 15 min were required to answer all questions. Participants completed the questionnaire voluntarily and were informed in advance of the purpose of the survey, the method used to answer questions, and the precautions taken with regard to the data. In the test, proven scales were used for the online measurements, which were completed independently by students on their computers or cell phones, thus emphasizing the principles of voluntary completion, the confidentiality of information, and anonymous completion.

A total of 856 questionnaires were collected, and after excluding questionnaires that featured excessively short answer times or invalid reverse answers, 765 questionnaires were valid, for an effective recovery rate of 89.37%. The ages of the participants ranged from 18 to 25 years, with a mean age of 20.24 ± 1.71 years. Among the participants, 355 (46.4%) were male, 410 (53.6%) were female; furthermore, 334 (43.7%) were studying the natural sciences, and 431 (56.3%) were studying the humanities or social sciences.

### 3.2 Measures

#### 3.2.1 Self-control scale

The self-control scale developed by Tangney et al. (2004) and revised by Tan and Guo (2008) was used. This scale consists of the five dimensions of impulse control, healthy habits, resisting temptation, focusing on work, and moderating recreation and features 19 items, e.g., “I can resist temptation very well” and “It is difficult for me to change bad habits”; this measure is scored on a 5-point scale (1 = “not at all,” 5 = “Completely”), in which context 15 items are reverse scored. The higher the total score, the better the individual's level of self-control. Research has shown that this scale exhibits good reliability and validity among Chinese college students (Li J. J. et al., 2023). Cronbach's alpha of this scale in this study was 0.880.

#### 3.2.2 Resilience scale

We measured resilience via the Connor-Davidson Resilience Scale (CD-RISC) (Campbell-Sills and Stein, 2007). This scale was revised by Ye et al. (2016) and consists of 10 questions. The scale was scored on a 5-point scale (0 = “never like this”, 4 = “often like this”), with a higher total score indicating that the individual is more mentally resilient. Research has shown that this scale exhibits good reliability and validity among Chinese college students (Liao et al., 2023). Cronbach's alpha of this scale in this study was 0.939.

#### 3.2.3 Positive emotions scale

We measured college students' positive emotions via the positive emotion dimension of the Scale of Positive and Negative Experience (SPANE). This scale was developed by Diener et al. (2009) and includes positive emotions (SPANE-P) and negative emotions (SPANE-N). The scale includes six question items rated on a 5-point scale (1 = “very little,” 5 = “very much”). The higher the score, the higher the individual's level of positive emotions. Research has shown that this scale exhibits good reliability and validity among Chinese college students (Fan et al., 2012). Cronbach's alpha of this scale in this study was 0.972.

#### 3.2.4 Learning engagement scale

A total of 17 questions were drawn from the learning engagement scale developed by Schaufeli et al. (2002) and revised by Fang et al. (2008), which included the dimensions of vigor, dedication, and concentration. This measure was rated on a 7-point scale (1 = “never happens,” 7 = “always happens”), with higher scores indicating higher levels of learning engagement. Research has shown that the scale exhibits good reliability and validity among Chinese college students (Wei, 2023). Cronbach's alpha of this scale in this study was 0.967.

### 3.3 Statistical analysis

Statistical analysis was performed using SPSS 26.0 and AMOS 26.0 software. The data analysis procedure was as follows:

First, a common method bias analysis was conducted with SPSS 26.0. Specifically, all items were subjected to Harman's one-factor test (unrotated exploratory factor analysis) (Podsakoff et al., 2003). If the variance explained by the first component, among all components with eigenvalues larger than 1, is < 40%, it suggests that there is no common method bias present in this study (Zhou and Long, 2004).

Second, the descriptive statistics, Pearson's correlation tests, and regression analyses for the four variables of self-control, resilience, positive emotions, and learning engagement were conducted using SPSS 26.0.

Third, the model fit indicators as well as the mediating effect were tested using AMOS26.0. We estimated the 95% confidence intervals of the mediating effect with 5,000 resamples.

## 4 Results

### 4.1 Common method bias test

Since all data in this study were from participants' self-reports, the Harman single factor test was used to conduct common method bias tests before analyzing the results. The results showed that there were eight factors with eigenvalues >1, and the variance of the first factor explained was 36.29%, less than the critical criterion of 40% (Zhou and Long, 2004), so there was no significant common method bias.

### 4.2 Descriptive statistics and correlation analysis among variables

The mean scores of self-control, resilience, positive emotions, and learning engagement were determined by correlation analysis, and the mean scores, standard deviations, and correlation coefficients of each variable are shown in Table 1. The results showed that self-control, resilience, positive emotions, and learning engagement were significantly and positively correlated.

**Table 1:** The description and the correlation of variables (*N* = 765).

	**M**	**SD**	**1**	**2**	**3**	**4**
1. SC	3.28	0.44	1			
2. RE	3.44	0.54	0.474^**^	1		
3. PE	3.61	0.78	0.477^**^	0.614^**^	1	
4. LE	4.41	0.75	0.479^**^	0.504^**^	0.483^**^	1

*N* = 765. ^*^p < 0.05, ^**^p < 0.01, and ^***^p < 0.001. SC, self-control; RE, resilience; PE, positive emotions; LE, learning engagement.

### 4.3 Regression analysis among variables

Regression analyses showed (as presented in Table 2) that self-control was a direct positive predictor of college students' resilience (β = 0.474, *p* < 0.001). Self-control and resilience positively predicted positive emotions (β = 0.241, *p* < 0.001; β = 0.500, *p* < 0.001). Self-control, resilience, and positive emotions positively predicted learning engagement (β = 0.262, *p* < 0.001; β = 0.258, *p* < 0.001; β = 0.200, *p* < 0.001). Furthermore, self-control was a direct positive predictor of college students' learning engagement (β = 0.479, *p* < 0.001).

**Table 2:** Results of the linear regression analysis.

**Dependent variable**	**Independent variable**	**R**	**Adjust R^2^**	**F**	**β**	**t**
RE		0.474	0.224	221.007^***^		
	SC				0.474	14.866^***^
PE		0.649	0.420	277.666^***^		
	SC				0.241	7.687^***^
	RE				0.500	15.973^***^
LE		0.593	0.349	137.449^***^		
	SC				0.262	7.600^***^
	RE				0.258	6.726^***^
	PE				0.200	5.199^***^
LE		0.479	0.228	227.095^***^		
	SC				0.479	15.070^***^

*N* = 765. ^*^p < 0.05, ^**^p < 0.01, and ^***^p < 0.001.

### 4.4 Intermediary analysis

This study used AMOS 26.0 to conduct structural equation model analysis with the goal of examining the mediating effects of resilience and positive emotion on the relationship between self-control and learning engagement. The fit index of the model was χ^2^/df = 4.986, GFI = 0.960, CFI = 0.972, NFI = 0.965, IFI = 0.972, TLI = 0.959, RMSEA = 0.072, and SRMR = 0.040, which met the requirements in terms of psychometrics.

**Figure 2:**
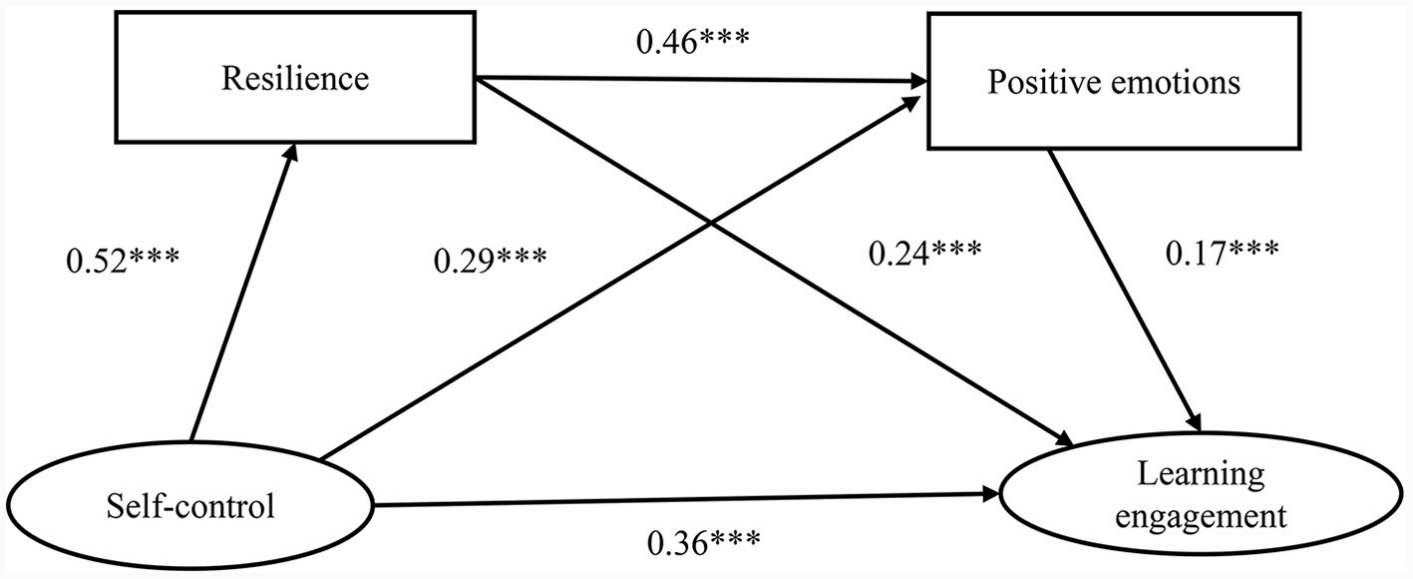
Chain-mediating effect of resilience and positive emotions. ^*^p < 0.05, ^**^p < 0.01, and ^***^p < 0.001.

Additionally, the mediating effects of each mediation path were examined using the bootstrap method (with 5,000 resamples) (Wen et al., 2004). The results are shown in Table 3 and Figure 2. These results suggest that resilience and positive emotions partially mediate the relationship between self-control and engagement in learning. Specifically, the mediating effect consisted of profile effects generated by the three pathways. None of the bootstrap 95% confidence intervals for the three indirect effects contained 0; therefore, all three indirect effects reached the level of significance. The total mediated effect value was 0.345, which accounted for 36.86% of the total effect value, and none of the 95% confidence intervals contained 0. The total mediated effect was thus significant. The direct effect value of self-control → learning engagement was 0.591, accounting for 63.14% of the total effect value, and none of the 95% confidence intervals contained 0. The direct effect was thus significant. The mediating effect of self-control → resilience → learning engagement was 0.201, accounting for 21.47% of the total effect value, and none of the 95% confidence intervals contained 0. The mediation effect was thus significant. The mediating effect of self-control → positive emotions → learning engagement mediation effect was 0.079, accounting for 8.44% of the total effect value, and none of the 95% confidence intervals contained 0. The mediating effect was thus significant. The mediating effect of self-control → resilience → positive emotions → learning engagement was 0.065, accounting for 6.94% of the total effect value, and none of the 95% confidence intervals contained 0. The chain-mediating effect was thus significant.

**Table 3:** Bootstrapping results of the mediating effect.

**Pathway**	**Point estimate**	**SE**	**95%CIs**	**Relative mediation effect**
SC → RE → LE	0.201	0.045	[0.117, 0.295]	21.47%
SC → PE → LE	0.079	0.022	[0.040, 0.127]	8.44%
SC → RE → PE → LE	0.065	0.018	[0.033, 0.104]	6.94%
SC → LE	0.591	0.081	[0.435, 0.748]	63.14%
Total indirect effect	0.345	0.051	[0.254, 0.451]	36.86%
Total effect	0.936	0.067	[0.806, 1.066]	100%

Bootstrap size = 5,000. CI, confidence interval.

## 5 Discussion

### 5.1 Mechanisms underlying the effect of self-control on learning engagement

These results show that there is a significant positive correlation between self-control and learning engagement, thus supporting hypothesis 1. This study found that self-control has a positive predictive effect on college students' learning engagement and that a higher level of self-control among college students helps increase their level of learning engagement, which is consistent with the results of previous studies (Yang, 2021). Students with higher self-control are able to maintain good study habits, avoid emotional disturbances, and invest more motivation and energy in their studies (Tangney et al., 2004; De Ridder et al., 2012). With the rapid development of the data era, cell phone cyberspace (such as hand games and live broadcasting) has gradually become the main field for college students to seek stimulation. College students who fail in self-control in the face of online temptation emerge with insufficient commitment to their studies (Antons et al., 2020; Sun et al., 2023). According to resource conservation theory, individuals with good self-control avoid excessive ego depletion and the loss of individual resources in the face of temptations such as cell phone networks, thus reducing their likelihood of self-control failures in subsequent tasks; such individuals thus perform better than the general population (Hobfoll, 2001; Dvorak and Simons, 2009). This finding suggests that students with higher levels of self-control are better able to resist external temptations and focus on their current learning activities (Steel, 2007). Thus, college students' level of self-control positively predicts their level of learning engagement.

### 5.2 The mediating role of resilience

The findings indicated that self-control significantly predicted resilience and that resilience significantly predicted learning engagement, which is more consistent with previous studies (Chen J. J. et al., 2021; Zheng and Xu, 2022). The mediation analysis showed that resilience mediated the relationship between self-control and learning engagement, thus supporting hypothesis 2. This finding is consistent with the self-control resource theory, which posits that individuals in a state of ego depletion exhibit impulsive decision-making due to the decline in self-control caused by insufficient resources and that resilience, as a form of positive psychological capital, can alleviate college students' depletion of self-control resources and increase their ability to engage in learning (Baumeister et al., 1998; Zheng and Xu, 2022). Researchers have found that college students' self-control positively predicts their resilience and that the level of resilience is strongly and positively related to their academic performance (Li and Li, 2013; Feng et al., 2022). Therefore, the present study demonstrates that resilience mediates the relationship between self-control and learning engagement.

### 5.3 The mediating role of positive emotions

The present study found that self-control positively and significantly predicted positive emotions and that positive emotions significantly and positively predicted learning engagement, results that are consistent with previous research (Bao et al., 2022). The mediation analysis showed that positive emotions mediated the relationship between self-control and learning engagement, thus verifying hypothesis 3. Previous studies have found that self-control positively predicts positive emotions; simultaneously, according to the extended construct theory of positive emotions, positive emotions such as pleasure and delight can increase students' motivation and enhance their engagement in learning (Fredrickson, 2001), thus motivating college students to approach their studies with a more positive attitude. Students with low self-control have poor study habits such as procrastination, which leads to weakened motivation, and when students are in a state of depletion, positive emotions can effectively promote the replenishment of their self-control resources, thus improving their self-control ability (Shen et al., 2021). This finding suggests that positive emotions have an alternative restorative effect on the depletion of self-control resources and that when individuals experience problems such as distraction from their learning due to external stressful environments, positive emotions can improve their attention and thinking qualities in the short term, thus leading to positive attitudes and behaviors, which can motivate them to invest more in the task of achieving their goals (Zhang and Wang, 2017; Hu et al., 2018; Liu et al., 2020).

### 5.4 The chain mediating effects of resilience and positive emotions

The present study found that resilience was significantly associated with positive emotions and that these factors had a chain mediating effect on the relationship between college students' self-control and engagement in learning, thus supporting hypothesis 4. The positive association between resilience and positive emotions as individual psychological resources confirmed the idea of the “resource gain spiral” posited by the conservation of resources theory (Hobfoll, 2011), which indicates that college students with high levels of resilience also have access to positive emotional resources. This is also consistent with existing research; for example, Diotaiuti et al. (2021a) studied athletes based on the resilience model proposed by Richardson (2002) and found positive associations between two different resilience types of resources. It was found that college students with greater resilience also have high levels of positive emotions, expanding the amount of individual resources they possess and motivating them to engage in learning under resource-rich conditions. However, self-control is a process that depletes individual resources, and when such resources are depleted, self-control fails (Park et al., 2016). At this point, resilience and positive emotions, as positive psychological resources available to individuals, can motivate individuals to recover from stressful situations and continue to engage in learning tasks (Cui et al., 2012). This finding corroborates the claim that positive emotions are a resource that resilient individuals can use to cope with distress and stress (Zautra et al., 2010). Highly mentally resilient individuals exhibit more positive emotional traits, can experience more positive emotions, and can effectively modulate the stimulation of positive emotions in cases of stress (Tugade and Fredrickson, 2004).

## 6 Main contributions and limitations

The theoretical significance of this study lies in the fact that it reveals the mechanism underlying the influence of self-control on learning engagement, thus improving our understanding of the influence of multiple individual factors and their interactions on college students' learning engagement. First, this study enriches previous research on the factors influencing college students' learning engagement and expands the research on the effects of self-control affecting learning engagement by exploring the mechanism of the relationship. Second, this study identified the resilience variable as a further mechanism underlying the relationship between self-control and learning engagement, thus highlighting countermeasures that can be used to reduce the negative impact of college students' self-control failure on learning engagement. Finally, based on the notion of the “resource gain spiral” posited by resource conservation theory, this study identifies resilience and positive emotions as chain mediating variables with the goal of exploring the effects of multiple key resources on learning engagement, thus verifying the interconnectedness and symbiosis among individual resources. The results of this study indicate that, first, teachers can play a leading role in the task of helping college students examine their self-control ability and that various teaching methods can be used to promote college students' learning engagement level according to their different roles of self-control, resilience, and positive emotions in learning engagement. Meanwhile, teachers should improve their teaching enthusiasm and devote themselves to classroom teaching with positive emotions, so as to stimulate the positive emotions of college students and enhance their learning engagement. Second, resilience can help college students deal with pressure and temptation by relying on positive emotions, which can improve their ability to cope with stressful situations in their studies and lives. Therefore, colleges and universities should regularly provide resilience training to enhance the positive emotions of college students with the goal of mitigating the negative impact of self-control failure on learning engagement. Finally, educators should regularly use the resilience scale, the positive emotions scale, and the interpersonal reactivity index scale to conduct timely tracking surveys of college students' psychological health and emotional state (Campbell-Sills and Stein, 2007; Diener et al., 2009; Diotaiuti et al., 2021b). Based on the results, targeted psychoeducational interventions will be provided to students with problems to maintain and improve their mental and emotional states.

However, this study also has the following limitations. (1) The cross-sectional design used in this study does not prove causality, and a tracking-type study can be conducted in the future to further confirm the findings of this research. (2) This study examines only the studies of university students in the Guangxi region of China. In the future, this research can be extended to the eastern and central regions of China with the goal of comparing the studies of university students in the central and western regions of China, which can provide support for the development of targeted measures that can narrow the quality gap in higher education in the central and western regions of the country.

## 7 Conclusion

With the conservation of resources theory and the extended construct theory of positive emotions applied, this study used a cross-sectional research design to explore the relationship between self-control and learning engagement as well as to reveal the mechanism of self-control's effect on learning engagement. Specifically, the findings indicated that there was a significant positive relationship between self-control and learning engagement among Chinese university students. We further tested the mediating roles of resilience and positive emotions in self-control and learning engagement. This mediation effect contains three mediating paths: the independent mediating effect of resilience, the independent mediating effect of positive emotions, and the chain mediating effect of resilience and positive emotions. This study reveals that Chinese college students' learning engagement is influenced by individual factors, including self-control, resilience, and positive emotions. Therefore, first, universities and teachers should actively carry out psychological training to enrich the individual psychological resources of college students so that they can make correct behavioral decisions in the face of pressure and temptation. Second, it is necessary for schools to construct a mental health education model oriented by the concept of positive psychology, carry out self-management and self-education for college students, enhance their goal and time management abilities, and help them form self-discipline habits so as to improve their self-control ability. Third, colleges and universities ought to improve the professional quality of college teachers, such as teaching methods and emotional management, as well as organize various forms of learning and skill competitions, while increasing the investment in hardware facilities, such as libraries and study rooms. In these ways, colleges and universities are able to provide college students with excellent teaching staff and a good learning atmosphere, thus increasing college students' learning engagement to improve the quality of college cultivation and achieve the purpose of enhancing the school's effectiveness.

## Data availability statement

The original contributions presented in the study are included in the article/Supplementary material, further inquiries can be directed to the corresponding author.

## Ethics statement

Ethical review and approval was not required for the study on human participants in accordance with the local legislation and institutional requirements. The studies were conducted in accordance with the local legislation and institutional requirements. The participants provided their written informed consent to participate in this study.

## Author contributions

Y-DY: Data curation, Software, Writing—original draft. C-LZ: Data curation, Funding acquisition, Investigation, Resources, Writing—review & editing. Z-QW: Data curation, Writing—original draft.
