# Supplementary material for: The relationship between self-control and learning engagement among Chinese college students: the chain mediating roles of resilience and positive emotions
Source: Front Psychol. 2024 Feb 20;15:1331691. doi: 10.3389/fpsyg.2024.1331691 (PMC10913274; doi:10.3389/fpsyg.2024.1331691)
Supplement: Supplementary file 2 [file Table_2.DOCX]

高校大学生学习情况调查

同学：

您好！该问卷仅用于学术研究，我们会对您所填写的信息进行保密。回答不分对错，每道题均为单选题，请您仔细填写每一道题目，结合自己的实际情况进行作答。您的参与对学术研究具有重要价值，感谢您的支持！

1. 您的性别：🞎男 🞎女
2. 您的年龄（请填写数字，如23）
3. 您所学专业为：🞎人文社科类 🞎自然科学类
4. 请您回忆过去一个月里的所作所为，并对一下感受的经历程度做出判断

| 1.积极的 | 非常少 | 比较少 | 有时 | 经常 | 非常多 |
| --- | --- | --- | --- | --- | --- |
| 2.好的 | 非常少 | 比较少 | 有时 | 经常 | 非常多 |
| 3.愉快的 | 非常少 | 比较少 | 有时 | 经常 | 非常多 |
| 4.幸福的 | 非常少 | 比较少 | 有时 | 经常 | 非常多 |
| 5.喜悦的 | 非常少 | 比较少 | 有时 | 经常 | 非常多 |
| 6.满足的 | 非常少 | 比较少 | 有时 | 经常 | 非常多 |

1. 请先根据您的真实想法，考虑是否符合您的实际情况，并选择相应的选项：

| 1.我能很好地抵制诱惑 | 完全不符合 | 不符合 | 不确定 | 符合 | 完全符合 |
| --- | --- | --- | --- | --- | --- |
| 2.对我来说改掉坏习惯是困难的 | 完全不符合 | 不符合 | 不确定 | 符合 | 完全符合 |
| 3.我是懒惰的 | 完全不符合 | 不符合 | 不确定 | 符合 | 完全符合 |
| 4.我会做一些能给自己带来快乐但对自己有害的事情 | 完全不符合 | 不符合 | 不确定 | 符合 | 完全符合 |
| 5.人们相信我能坚持行动计划 | 完全不符合 | 不符合 | 不确定 | 符合 | 完全符合 |
| 6.对我来说，早上起床是件困难的事 | 完全不符合 | 不符合 | 不确定 | 符合 | 完全符合 |
| 7.大家说我是冲动的 | 完全不符合 | 不符合 | 不确定 | 符合 | 完全符合 |
| 8.我太能花钱了 | 完全不符合 | 不符合 | 不确定 | 符合 | 完全符合 |
| 9.我会因情感而激动的不能自持 | 完全不符合 | 不符合 | 不确定 | 符合 | 完全符合 |
| 10.我做的很多事情都是因为一时冲动 | 完全不符合 | 不符合 | 不确定 | 符合 | 完全符合 |
| 11.大家说我有钢铁般的自制力 | 完全不符合 | 不符合 | 不确定 | 符合 | 完全符合 |
| 12.有时我会被有乐趣的事情干扰我而不能按时完成任务 | 完全不符合 | 不符合 | 不确定 | 符合 | 完全符合 |
| 13.我难以集中注意力 | 完全不符合 | 不符合 | 不确定 | 符合 | 完全符合 |
| 14.我能为了一个长远目标高效地工作 | 完全不符合 | 不符合 | 不确定 | 符合 | 完全符合 |
| 15.有时我会忍不住去做一些事情，即使我知道那样做是错误 | 完全不符合 | 不符合 | 不确定 | 符合 | 完全符合 |
| 16.我常常考虑不周就付诸行动 | 完全不符合 | 不符合 | 不确定 | 符合 | 完全符合 |
| 17.我太容易发脾气 | 完全不符合 | 不符合 | 不确定 | 符合 | 完全符合 |
| 18.我经常打扰别人 | 完全不符合 | 不符合 | 不确定 | 符合 | 完全符合 |
| 19.我有时会饮酒（或上网）过度 | 完全不符合 | 不符合 | 不确定 | 符合 | 完全符合 |

1. 请先根据您的真实想法，考虑是否符合您的实际情况，并选择相应的选项：

| 1.当发生变化时，我能够适应 | 从不这样 | 很少这样 | 有时这样 | 经常这样 | 总是这样 |
| --- | --- | --- | --- | --- | --- |
| 2.不管发生什么事情，我都能处理 | 从不这样 | 很少这样 | 有时这样 | 经常这样 | 总是这样 |
| 3.面对难题时，我试着去看事情幽默的一面 | 从不这样 | 很少这样 | 有时这样 | 经常这样 | 总是这样 |
| 4.克服压力让我变得坚强 | 从不这样 | 很少这样 | 有时这样 | 经常这样 | 总是这样 |
| 5.在生病、受伤或苦难之后，我很容易就恢复过来 | 从不这样 | 很少这样 | 有时这样 | 经常这样 | 总是这样 |
| 6.纵然有阻碍，我相信我能实现我的目标 | 从不这样 | 很少这样 | 有时这样 | 经常这样 | 总是这样 |
| 7.压力之下，我仍然能集中精神思考问题 | 从不这样 | 很少这样 | 有时这样 | 经常这样 | 总是这样 |
| 8.我不会因为失败就容易气馁 | 从不这样 | 很少这样 | 有时这样 | 经常这样 | 总是这样 |
| 9.在处理生命中的挑战和困难时，我认为自己是个坚强的人 | 从不这样 | 很少这样 | 有时这样 | 经常这样 | 总是这样 |
| 10.我能处理一些不愉快或痛苦的感觉，如悲伤、害怕和生气 | 从不这样 | 很少这样 | 有时这样 | 经常这样 | 总是这样 |

1. 下面是关于学习上的一些问题，请选择与自己相符合的选项：1=“从来不发生”；2=“基本不发生，大约一年1-2次，甚至更少”；3=“很少发生，大约一月发生1次甚至更少”；4=“有时发生，大约一个月2-4次”；5=“经常发生，大约一周发生1次”；6=“非常普遍，大约一周2次甚至更多”；7=“总是发生，大约一天发生1次”

|  | 1  从不 | 2  几乎不 | 3  很少 | 4  有时 | 5  经常 | 6  非常普遍 | 7  总是 |
| --- | --- | --- | --- | --- | --- | --- | --- |
| 1.学习时，我感到精力充沛 | 1  从不 | 2  几乎不 | 3  很少 | 4  有时 | 5  经常 | 6  非常普遍 | 7  总是 |
| 2.学习时，我浑身有力而且干劲十足 | 1  从不 | 2  几乎不 | 3  很少 | 4  有时 | 5  经常 | 6  非常普遍 | 7  总是 |
| 3.早晨一起床，我就乐意去学习 | 1  从不 | 2  几乎不 | 3  很少 | 4  有时 | 5  经常 | 6  非常普遍 | 7  总是 |
| 4.我能持续学习很长时间，中间不需要休息 | 1  从不 | 2  几乎不 | 3  很少 | 4  有时 | 5  经常 | 6  非常普遍 | 7  总是 |
| 5.学习时，即使精神疲劳，我也能很快恢复 | 1  从不 | 2  几乎不 | 3  很少 | 4  有时 | 5  经常 | 6  非常普遍 | 7  总是 |
| 6.即使学习不顺利，我也毫不气馁，能够坚持不懈 | 1  从不 | 2  几乎不 | 3  很少 | 4  有时 | 5  经常 | 6  非常普遍 | 7  总是 |
| 7.我发现学习目的明确，而且很有意义 | 1  从不 | 2  几乎不 | 3  很少 | 4  有时 | 5  经常 | 6  非常普遍 | 7  总是 |
| 8.我对学习充满热情 | 1  从不 | 2  几乎不 | 3  很少 | 4  有时 | 5  经常 | 6  非常普遍 | 7  总是 |
| 9.学习激发我的灵感 | 1  从不 | 2  几乎不 | 3  很少 | 4  有时 | 5  经常 | 6  非常普遍 | 7  总是 |
| 10.我因我的学习而感到自豪 | 1  从不 | 2  几乎不 | 3  很少 | 4  有时 | 5  经常 | 6  非常普遍 | 7  总是 |
| 11.我发现学习富有挑战性 | 1  从不 | 2  几乎不 | 3  很少 | 4  有时 | 5  经常 | 6  非常普遍 | 7  总是 |
| 12.学习时，我感到时间过的很快 | 1  从不 | 2  几乎不 | 3  很少 | 4  有时 | 5  经常 | 6  非常普遍 | 7  总是 |
| 13.学习时，我忘了周围的一切 | 1  从不 | 2  几乎不 | 3  很少 | 4  有时 | 5  经常 | 6  非常普遍 | 7  总是 |
| 14.全身心投入学习时，我感到很快乐 | 1  从不 | 2  几乎不 | 3  很少 | 4  有时 | 5  经常 | 6  非常普遍 | 7  总是 |
| 15.我沉浸在学习中 | 1  从不 | 2  几乎不 | 3  很少 | 4  有时 | 5  经常 | 6  非常普遍 | 7  总是 |
| 16.学习时，我心里只想着学习 | 1  从不 | 2  几乎不 | 3  很少 | 4  有时 | 5  经常 | 6  非常普遍 | 7  总是 |
| 17.我难以放下手中的学习 | 1  从不 | 2  几乎不 | 3  很少 | 4  有时 | 5  经常 | 6  非常普遍 | 7  总是 |
